# Supplementary material for: Complete representation of a tapeworm genome reveals chromosomes capped by centromeres, necessitating a dual role in segregation and protection
Source: BMC Biol. 2020 Nov 9;18:165. doi: 10.1186/s12915-020-00899-w (PMC7653826; doi:10.1186/s12915-020-00899-w)
Supplement: Supplementary file 3 — Additional file 3: Figure S1. Mitochondrial genome. The 13,919 bp Hymenolepis microstoma mitochondrial genome was re-assembled from both short and long-read data, yielding over 1000x coverage. The new assembly resolved the full length of a region involving a tandemly repeated 32 bp motif (cf. GenBank accession AP017665.1). This region is identified as one of three origins of replication-heavy strand (OH-a) by MITOS [61] and an adjacent hairpin-loop region as the origin of replication-light strand (OL). Gene order of ribosomal and protein-coding genes is consistent with the hypothesized ground-plan for the mitogenomes of parasitic flatworms as is the absence of the atp8 gene [89]. [file 12915_2020_899_MOESM3_ESM.pdf]

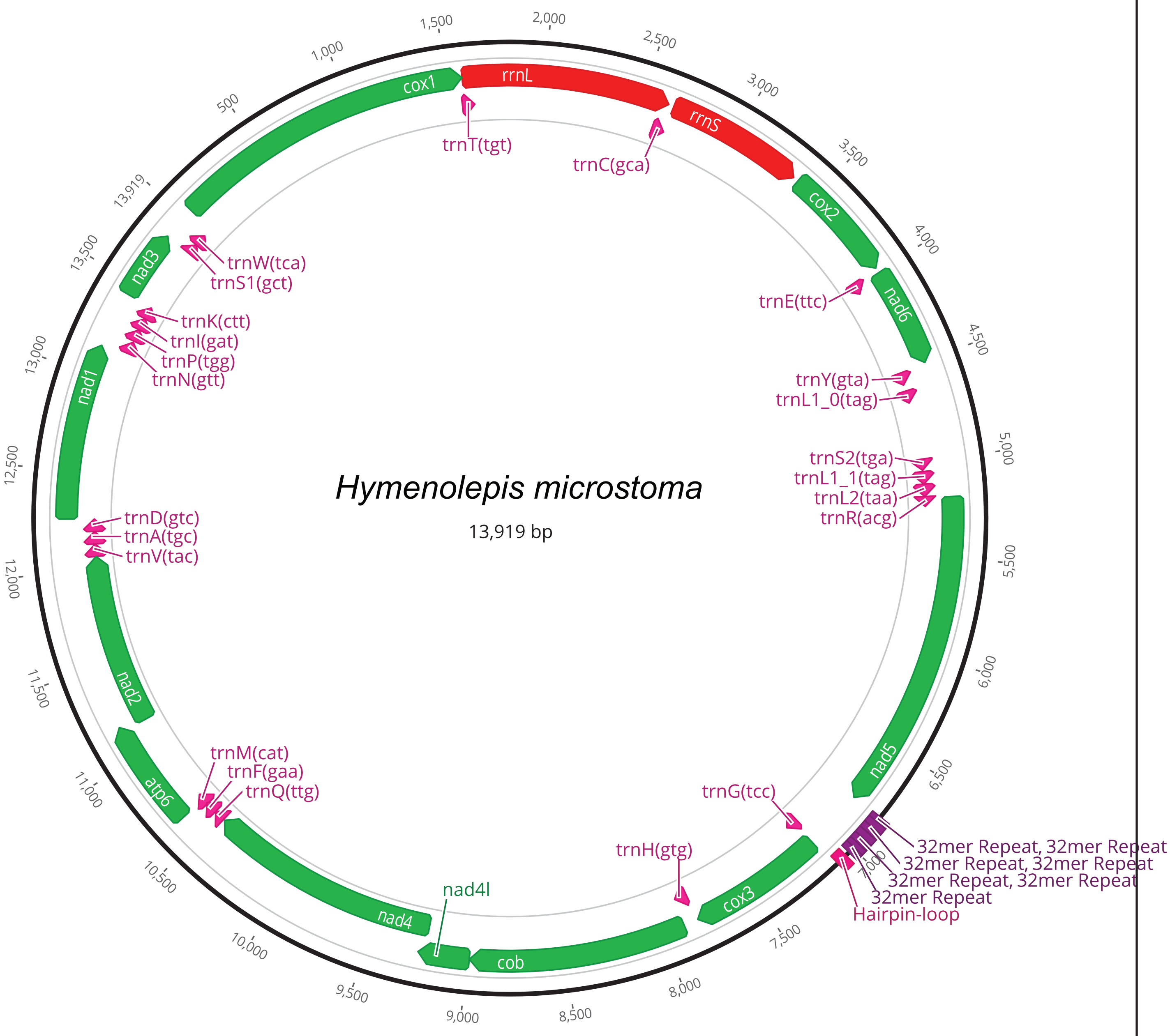

Supplementary Fig. S1. Mitochondrial genome. The 13,919 bp *Hymenolepis microstoma* mitochondrial genome was re-assembled from both short and long-read data, yielding over 1000x coverage. The new assembly resolved the full length of a region involving a tandemly repeated 32 bp motif (cf. GenBank accession AP017665.1). This region is identified as one of three origins of replication-heavy strand (OH-a) by MITOS [59] and an adjacent hairpin-loop region as the origin of replication-light strand (OL). Gene order of ribosomal and protein-coding genes is consistent with the hypothesized ground-plan for the mitogenomes of parasitic flatworms as is the absence of the atp8 gene [67].
