# Supplementary material for: Complete representation of a tapeworm genome reveals chromosomes capped by centromeres, necessitating a dual role in segregation and protection
Source: BMC Biol. 2020 Nov 9;18:165. doi: 10.1186/s12915-020-00899-w (PMC7653826; doi:10.1186/s12915-020-00899-w)
Supplement: Supplementary file 4 — Additional file 4: Figure S2. Repeat hotspots. Chromosomal positions of paralogous gene arrays. Abbreviations: ABCB: ATP binding cassette subfamily B; Akr1b4: Aldo keto reductase family 1 member B4; AP: Alkaline phosphatase; AQP: Aquaporin 4; CREBBP: CREB binding protein; DYNLL: Dynein light chain; EiF2c: Eukaryotic translation initiation factor 2c; ENPP: Ectonucleotide pyrophosphatase:phosphodiesterase; EP45: Estrogen regulated protein EP45; GST: Glutathione S transferase; H3: Histone H3; HSP: heat shock protein; hypo: hypothetical protein; MVP: Major vault protein; PARP: Poly [ADP-ribose] polymerase; PiT: Phosphate transporter; PNP: Purine nucleoside phosphorylase; PP2A: Serine:threonine protein phosphatase 2A; PURA: PUR alpha protein; USP: Universal stress protein; RAD51: DNA repair protein RAD51 homolog; SLC22: Solute carrier family 33; TSP: Tetraspanin; TXN: Thioredoxin; ZNF: zinc finger protein. [file 12915_2020_899_MOESM4_ESM.pdf]

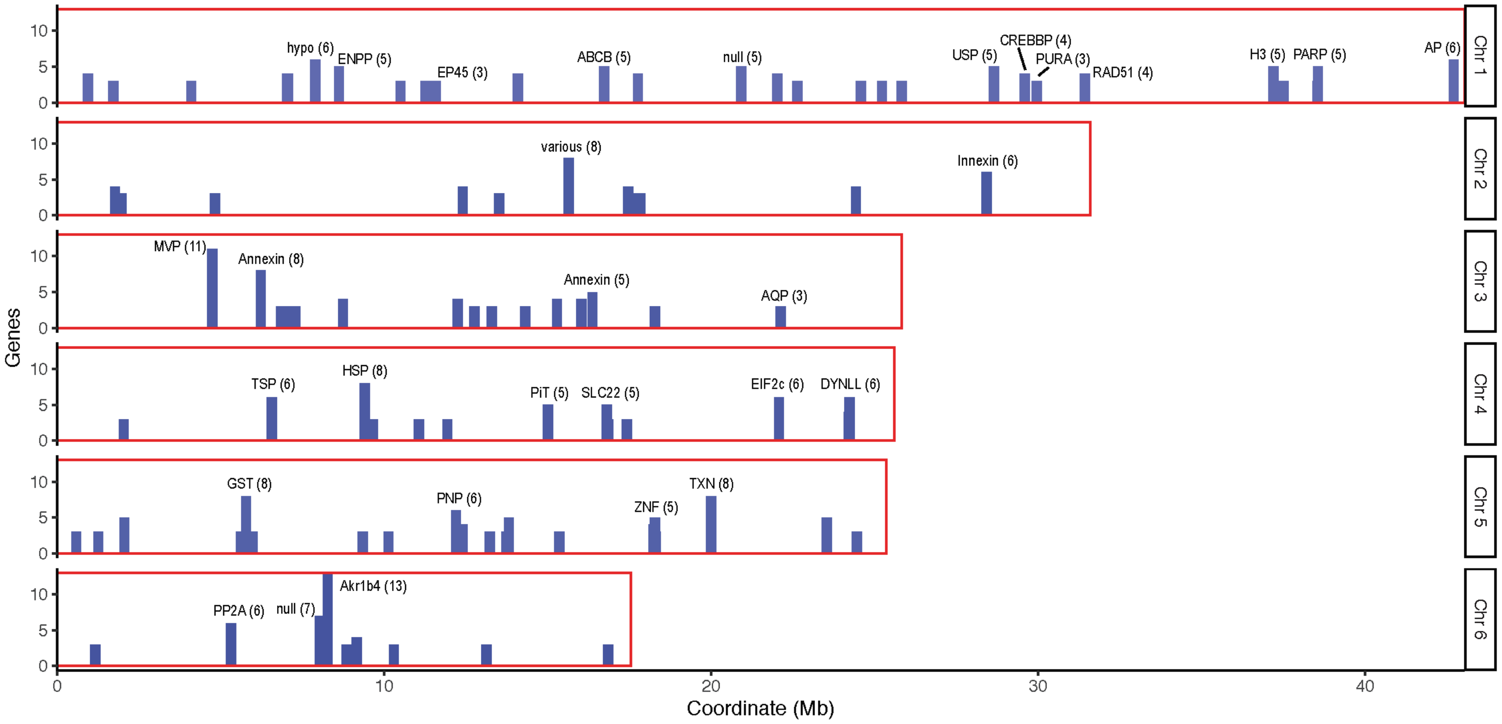

Supplementary Fig. S2. Repeat hotspots. Chromosomal positions of paralogous gene arrays. Abbreviations: ABCB: ATP binding cassette subfamily B; Akr1b4: Aldo keto reductase family 1 member B4; AP: Alkaline phosphatase; AQP: Aquaporin 4; CREBBP: CREB binding protein; DYNLL: Dynein light chain; EiF2c: Eukaryotic translation initiation factor 2c; ENPP: Ectonucleotide pyrophosphatase:phosphodiesterase; EP45: Estrogen regulated protein EP45; GST: Glutathione S transferase; H3: Histone H3; HSP: heat shock protein; hypo: hypothetical protein; MVP: Major vault protein; PARP: Poly [ADP-ribose] polymerase; PiT: Phosphate transporter; PNP: Purine nucleoside phosphorylase; PP2A: Serine:threonine protein phosphatase 2A; PURA: PUR alpha protein; USP: Universal stress protein; RAD51: DNA repair protein RAD51 homolog; SLC22: Solute carrier family 33; TSP: Tetraspanin; TXN: Thioredoxin; ZNF: zinc finger protein.
