# Supplementary material for: Complete representation of a tapeworm genome reveals chromosomes capped by centromeres, necessitating a dual role in segregation and protection
Source: BMC Biol. 2020 Nov 9;18:165. doi: 10.1186/s12915-020-00899-w (PMC7653826; doi:10.1186/s12915-020-00899-w)
Supplement: Supplementary file 5 — Additional file 5: Figure S3. Comparison of differentially expressed genes estimated from RNA-seq counts aligned to the v2 and v3 assemblies and gene models. Plots of log2-fold change show highly linear relationships across all sample comparisons, corroborating previous findings [8]. Only 11 genes (yellow), all with small fold-change values, were found to reverse directionality between assembly versions. [file 12915_2020_899_MOESM5_ESM.pdf]

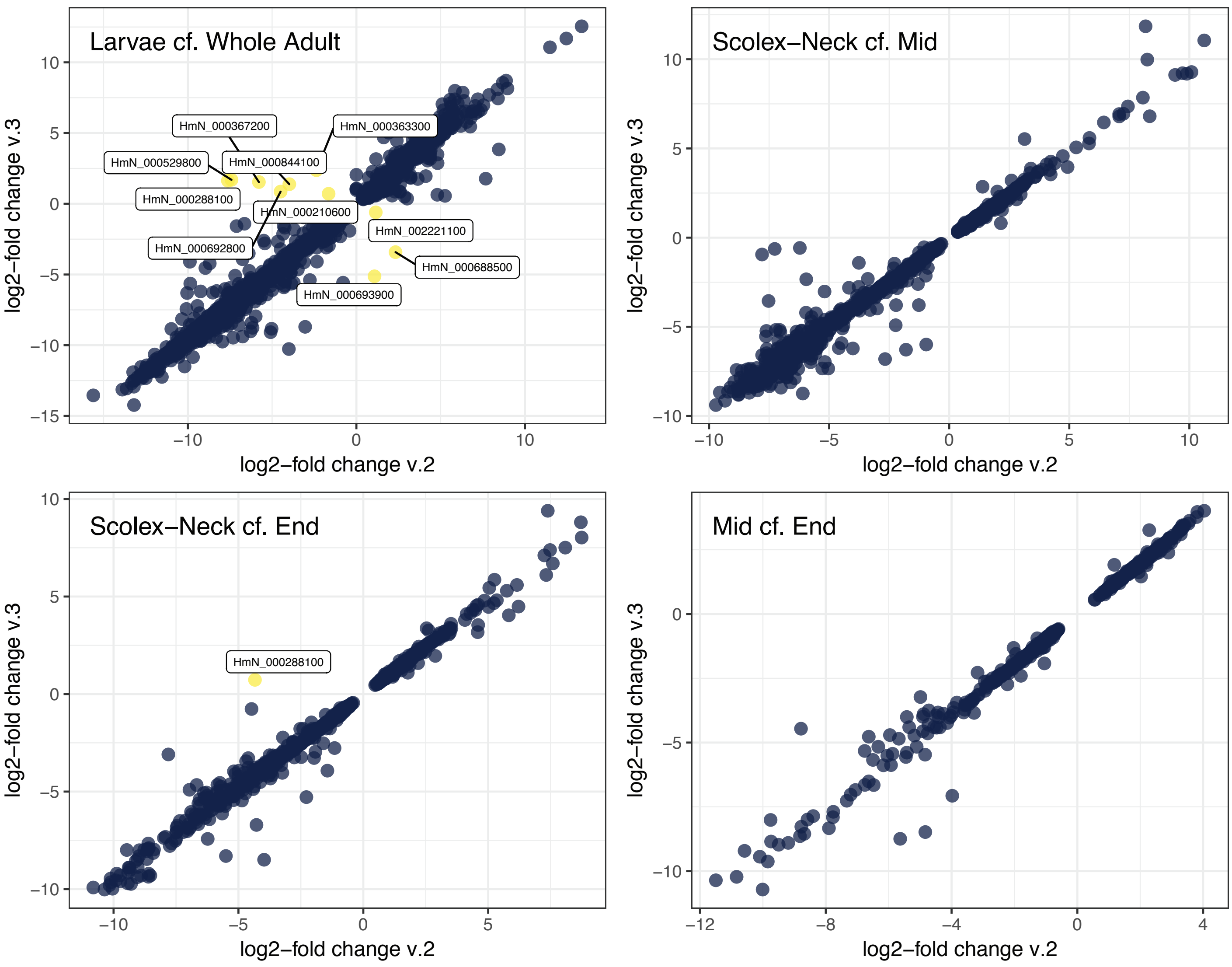

Supplementary Fig. S3. Comparison of differentially expressed genes estimated from RNA-seq counts aligned to the v2 and v3 assemblies. Plots of log2-fold change show highly linear relationships across all sample comparisons, corroborating previous findings [8]. Only 11 genes (yellow), all with small fold-change values, were found to reverse directionality between assembly versions.
