# Supplementary material for: Complete representation of a tapeworm genome reveals chromosomes capped by centromeres, necessitating a dual role in segregation and protection
Source: BMC Biol. 2020 Nov 9;18:165. doi: 10.1186/s12915-020-00899-w (PMC7653826; doi:10.1186/s12915-020-00899-w)
Supplement: Supplementary file 6 — Additional file 6: Figure S4. Comparison of RNA-seq sample counts against the v2 and v3 assemblies and gene models. Principle component analyses (A) show tight clustering of sample replicates based on counts using both assemblies, while in the v3 (right) the Larvae, Scolex-Neck and Whole Adult samples are arrayed only along PC1, with the transcriptome of the Scolex-Neck mid-way between those of the Larvae and Whole Adult samples. The Mid and End samples are further differentiated from the other samples along PC2. Heatmap clustering (B) shows that the transcriptome of the Scolex-Neck region is more similar to that of mid-metamorphose larvae than to middle or end regions of the adult worm, as discussed in [8]. [file 12915_2020_899_MOESM6_ESM.pdf]

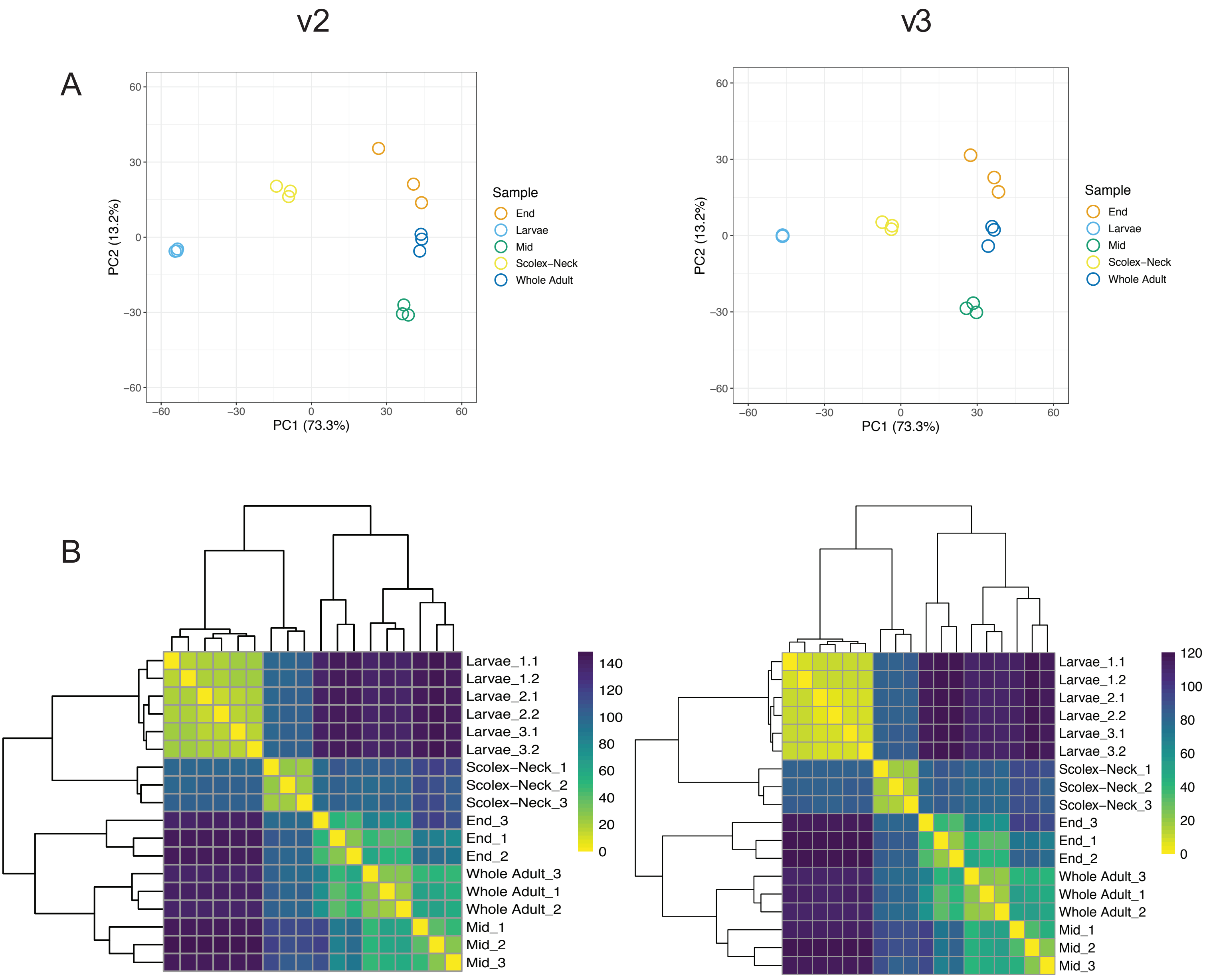

Supplementary Fig. S4. Comparison of RNA-seq sample counts against the v2 and v3 assemblies and gene models. Principle component analyses (**A**) show tight clustering of sample replicates based on counts using both assemblies, while in the v3 (right) the Larvae, Scolex-Neck and Whole Adult samples are arrayed only along PC1, with the transcriptome of the Scolex-Neck mid-way between those of the Larvae and Whole Adult samples. The Mid and End samples are further differentiated from the other samples along PC2. Heatmap clustering (**B**) shows that the transcriptome of the Scolex-Neck region is more similar to that of mid-metamorphose larvae than to middle or end regions of the adult worm, as discussed in [8].
