# Supplementary material for: Complete representation of a tapeworm genome reveals chromosomes capped by centromeres, necessitating a dual role in segregation and protection
Source: BMC Biol. 2020 Nov 9;18:165. doi: 10.1186/s12915-020-00899-w (PMC7653826; doi:10.1186/s12915-020-00899-w)
Supplement: Supplementary file 7 — Additional file 7: Figure S5. Optical map contigs aligned to the genome assembly of the rRNA repeat array. Five contigs from the optical map are shown with the segment that aligns to the sequenced repeat indicated by coloured bars. The largest map contig (arrow) represents one haplotype containing the rRNA tandem repeat (pink bar) as well as the left (blue bar) and right (yellow bar) flanking regions. Other optical map contigs either contain the repeat together with either 5’ or 3’ flanking region, and likely represent an alternative haplotype, or have an insufficient amount of unique sequence to unambiguously determine their position within the repeat array. [file 12915_2020_899_MOESM7_ESM.pdf]

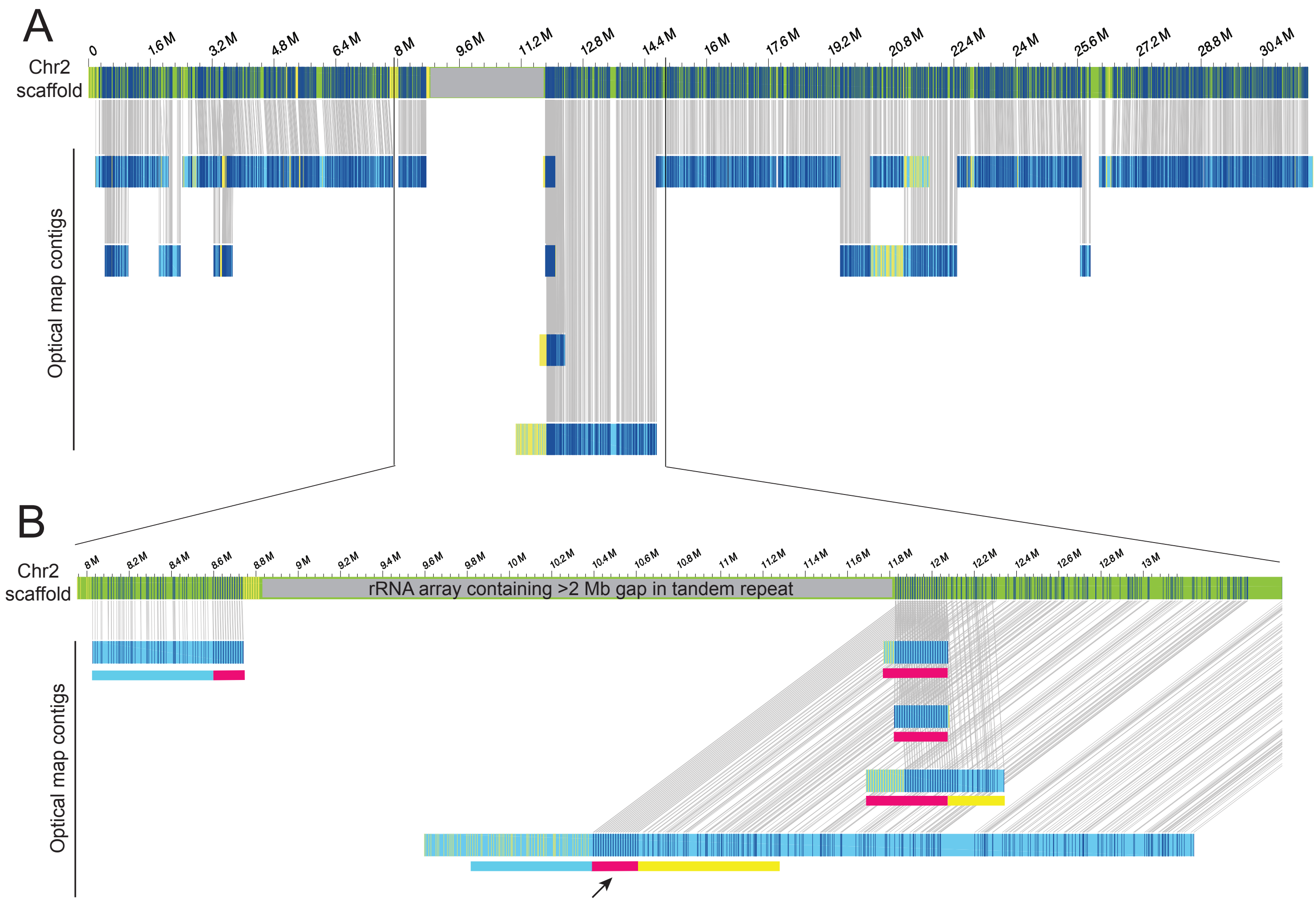

Supplementary Fig. S5. Optical map contigs aligned to the genome assembly of the rRNA repeat array. Five contigs from the optical map are shown with the segment that aligns to the sequenced repeat indicated by coloured bars. The largest map contig (arrow) represents one haplotype containing the rRNA tandem repeat (pink bar) as well as the left (blue bar) and right (yellow bar) flanking regions. Other optical map contigs either contain the repeat together with either 5' or 3' flanking region, and likely represent an alternative haplotype, or have an insufficient amount of unique sequence to unambiguously determine their position within the repeat array.
