# Supplementary material for: Complete representation of a tapeworm genome reveals chromosomes capped by centromeres, necessitating a dual role in segregation and protection
Source: BMC Biol. 2020 Nov 9;18:165. doi: 10.1186/s12915-020-00899-w (PMC7653826; doi:10.1186/s12915-020-00899-w)
Supplement: Supplementary file 8 — Additional file 8: Figure S6. Whole genome optical maps aligned to v3 assembly. Circled regions show where optical map data indicate alternative haplotypic versions. Regions labelled A and B, together with the rRNA array, represent the largest repeat regions where haplotype differences could account for visible length differences in sister chromatids (see text). Chromosomes are numbered and the positions of the telomeric repeats indicated by red dots. [file 12915_2020_899_MOESM8_ESM.pdf]

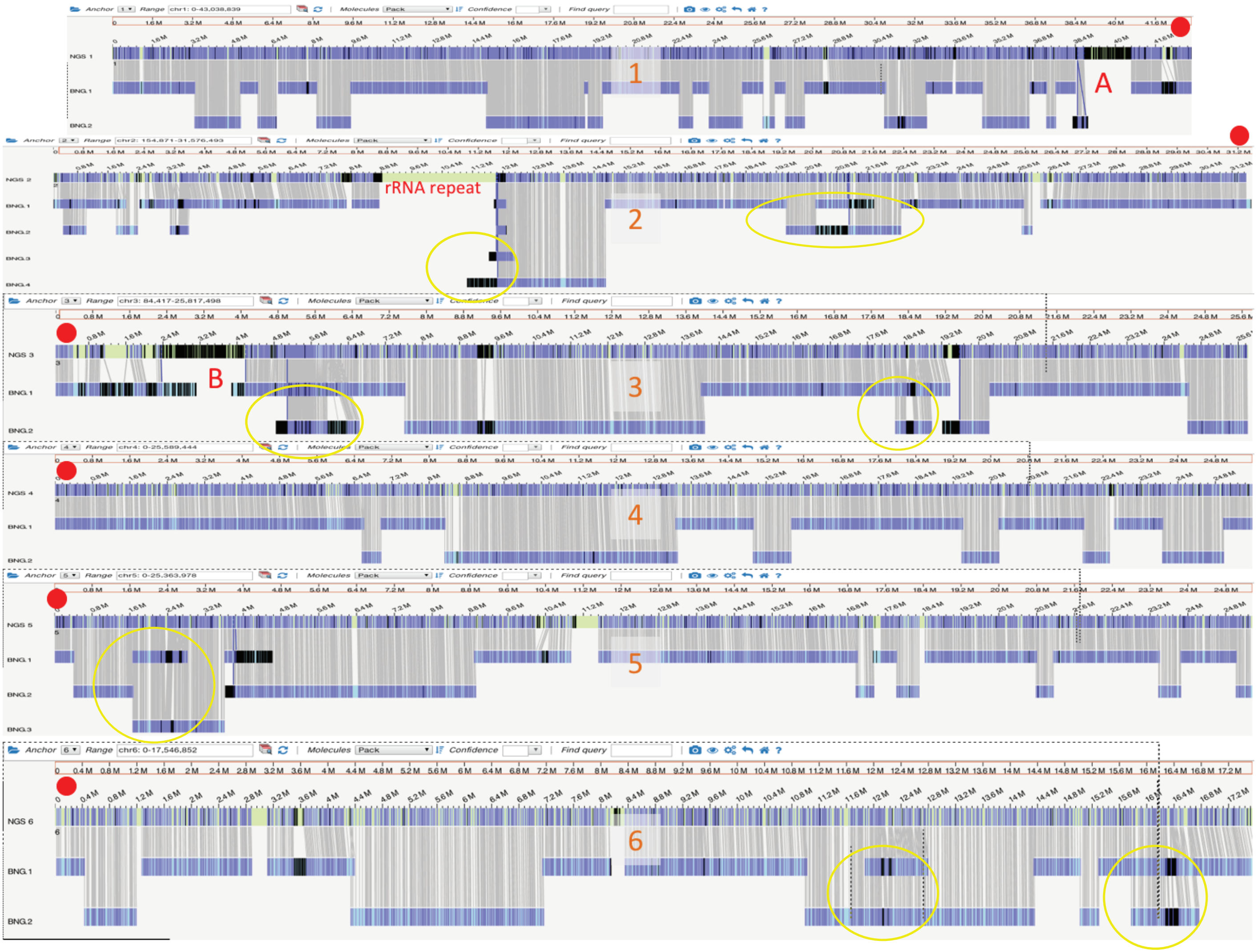

Supplementary Fig. S6. Whole genome optical maps aligned to v3 assembly. Circled regions show where optical map data indicate alternative haplotypic versions. Regions labelled A and B, together with the rRNA array, represent the largest repeat regions where haplotype differences could account for visible length differences in sister chromatids (see text). Chromosomes are numbered and the positions of the telomeric repeats indicated by red dots.
