# Supplementary material for: Complete representation of a tapeworm genome reveals chromosomes capped by centromeres, necessitating a dual role in segregation and protection
Source: BMC Biol. 2020 Nov 9;18:165. doi: 10.1186/s12915-020-00899-w (PMC7653826; doi:10.1186/s12915-020-00899-w)
Supplement: Supplementary file 9 — Additional file 9: Figure S7. Alignment of the N-terminal regions encoded by a tandem array of micro-exons genes located on Chr 6. The shared amino acid motif (consensus MRLFILLCFAVTLWAC) indicates that this gene array evolved through tandem duplication. [file 12915_2020_899_MOESM9_ESM.pdf]

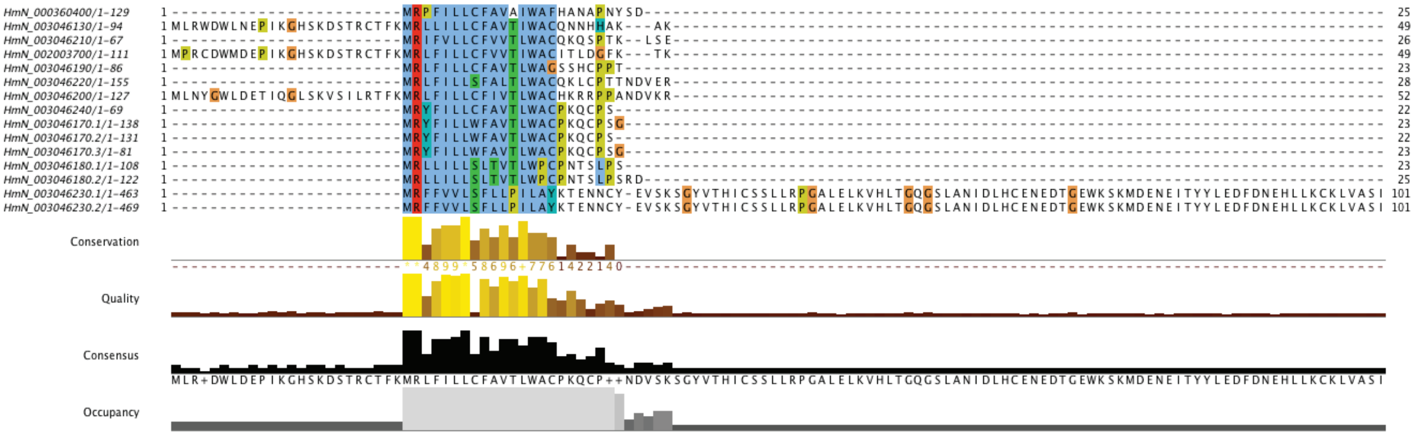

Supplementary Fig. S7. Alignment of the N-terminal regions encoded by a tandem array of micro-exons genes located on chromosome 6. The shared amino acid motif (consensus MRLFILLCFAVTLWAC) indicates that this gene array evolved through tandem duplication.
