# Supplementary material for: Complete representation of a tapeworm genome reveals chromosomes capped by centromeres, necessitating a dual role in segregation and protection
Source: BMC Biol. 2020 Nov 9;18:165. doi: 10.1186/s12915-020-00899-w (PMC7653826; doi:10.1186/s12915-020-00899-w)
Supplement: Supplementary file 10 — Additional file 10: Figure S8. Spliced leader trans-splicing. (A) Clustering of sequences soft clipped from aligned RNA-seq reads. The most abundant clusters represent known (E. multilocularis, S. mansoni) or candidate (H. microstoma) splice leader (SL) sequences which are given in the table below. (B) The prevalence of trans-splicing in different life stages and regions of the adult worm. Genes were considered trans-spliced if > 10 SL reads (SL1, SL2 or SL3) aligned across all libraries analysed. Of these genes, plot represents instances of at least one SL read aligning in each sample. Note that there are 5x as many genes trans-spliced in larvae than in the adult samples. Three replicates per sample. (C) An example of a gene (HmN_000032200) that is trans-spliced in larval but not adult samples, visualised using Apollo. Left: track 1 shows a coverage plot of all aligned reads; track 2 represents alignments of uniquely-mapping soft-clipped reads (soft clipping represented by a thick blue bar at the end of the read). Arrow indicates accumulation of soft clipped reads at proposed SL-acceptor site. Right: Coverage plots of all aligned reads in three larval and three adult libraries. Arrows indicate proposed SL-acceptor sites present in the larval but not adult libraries. (D) Venn diagram of trans-spliced orthogroups shared between parasitic flatworms. [file 12915_2020_899_MOESM10_ESM.pdf]

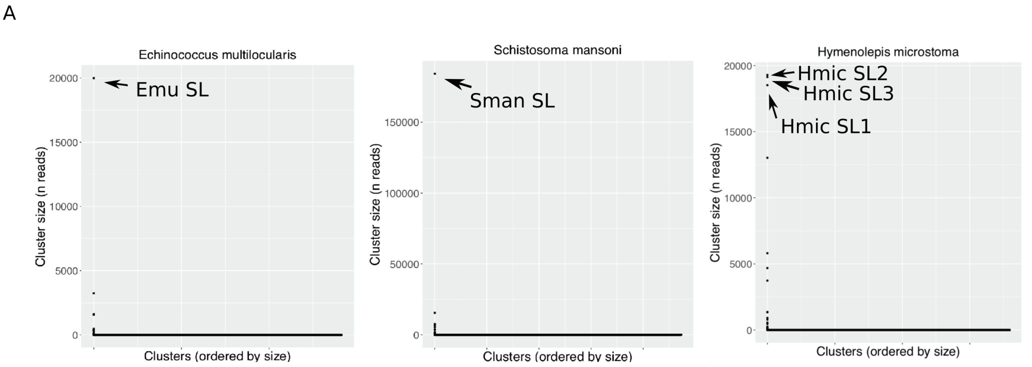

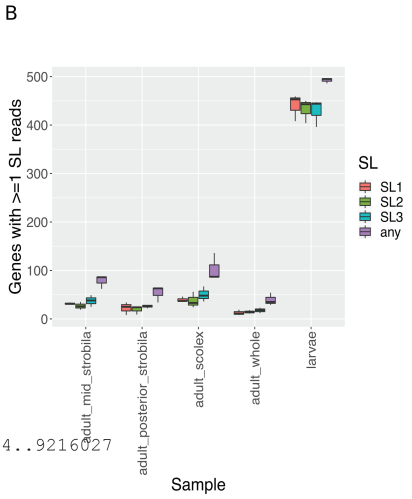

SL	Sequence	Genomic location
Hmic SL1	CGGTCTTACCATAAAACTTGT**ATG**	HMN_01_pilon:9215213..9215236, HMN_01_pilon:9216004..9216027
Hmic SL2	CCGGTCTTACCTTGCAATTTTTGT**ATG**	HMN_03_pilon:20837621..20837647
Hmic SL3	AATCGGTCTTACTGTACTAACTTGT**ATG**	HMN_01_pilon:5649152..5649179
Emu SL	TCGGTCCTTACCTTGCAGTTTTGT**ATG**	67 loci on 8 contigs/scaffolds (of which 25 within EmW_scaffold_01:16924000..16966000)
Sman SL	CACGGTTTTACTCTTGTGATTTGTTGC**ATG**	118 loci on 5 scaffolds (of which 109 within SM_V7_6:6847000..6942300)

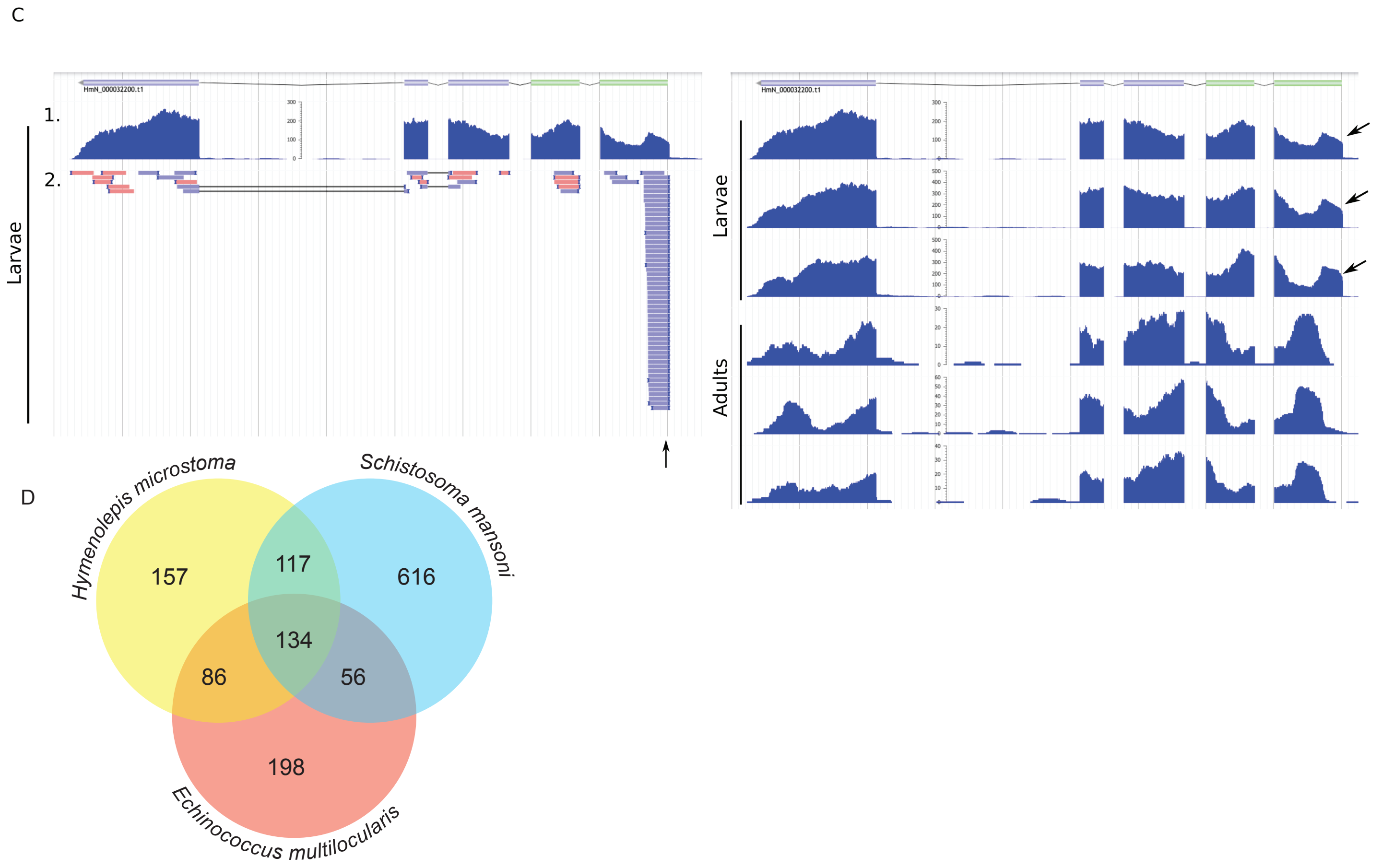

Supplementary Fig. S8. Spliced leader trans-splicing. (**A**) Clustering of sequences soft clipped from aligned RNAseq reads. The most abundant clusters represent known (*E. multilocularis*, *S. mansoni*) or candidate (*H. microstoma*) splice leader (SL) sequences which are given in the table below. N.B. SL sequences represent the soft-clipped trans-spliced donor sequence and do not represent full-length genomic SL sequences. (**B**) Prevalence of trans-splicing in different life stages and regions of the adult worm. Genes were considered trans-spliced if >10 SL reads (SL1, SL2 or SL3) aligned across all libraries analysed. Of these genes, plots represent instances of at least one SL read aligning in each sample. Note that there are almost 5x the number of gene models trans-spliced in larvae than in adult samples. Three replicates per sample. (**C**) An example of a gene (HmN_000032200) that is trans-spliced in larval, but not adult, samples visualised using Apollo. Left: track 1 shows a coverage plot of all aligned reads; track 2 represent alignments of uniquely-mapping soft-clipped reads (soft-clipping represented by a thick blue bar at the end of the read). Arrow indicates accumulation of soft-clipped reads at proposed SL-acceptor site. Right: Coverage plots of all aligned reads in three larval and three adult libraries. Arrows indicate proposed SL-acceptor sites present in the larval but not adult libraries. (**D**) Venn diagram of trans-spliced orthogroups shared between parasitic flatworms.
