# Supplementary material for: Complete representation of a tapeworm genome reveals chromosomes capped by centromeres, necessitating a dual role in segregation and protection
Source: BMC Biol. 2020 Nov 9;18:165. doi: 10.1186/s12915-020-00899-w (PMC7653826; doi:10.1186/s12915-020-00899-w)
Supplement: Supplementary file 11 — Additional file 11: Figure S9. Multiple alignment of the terminal centromeric repeats of each chromosome. 26 consecutive repeat copies were taken from a single location at the end of each of the six chromosomes in turn and aligned in order (top 26 = Chr1, next 26 = Chr2 etc.). Strong conservation of the 179mer centromeric repeat is seen across all chromosomes except Chr2 which shows a second novel repeat type. However, searching within the whole of the Chr2 repeat array shows that the ‘canonical’ 179mer observed in the other aligned reads is found with 100% coverage and identity. The terminal array on Chr2 is also much larger than those of the other chromosomes and is interspersed with various other repeats not shown here. Full assembly of the Chr2 terminal array is not resolvable without longer sequencing reads. Notably, when the centrosomal repeat arrays are oriented at the same end of each chromosome their sequences are found to be in alignment. [file 12915_2020_899_MOESM11_ESM.pdf]

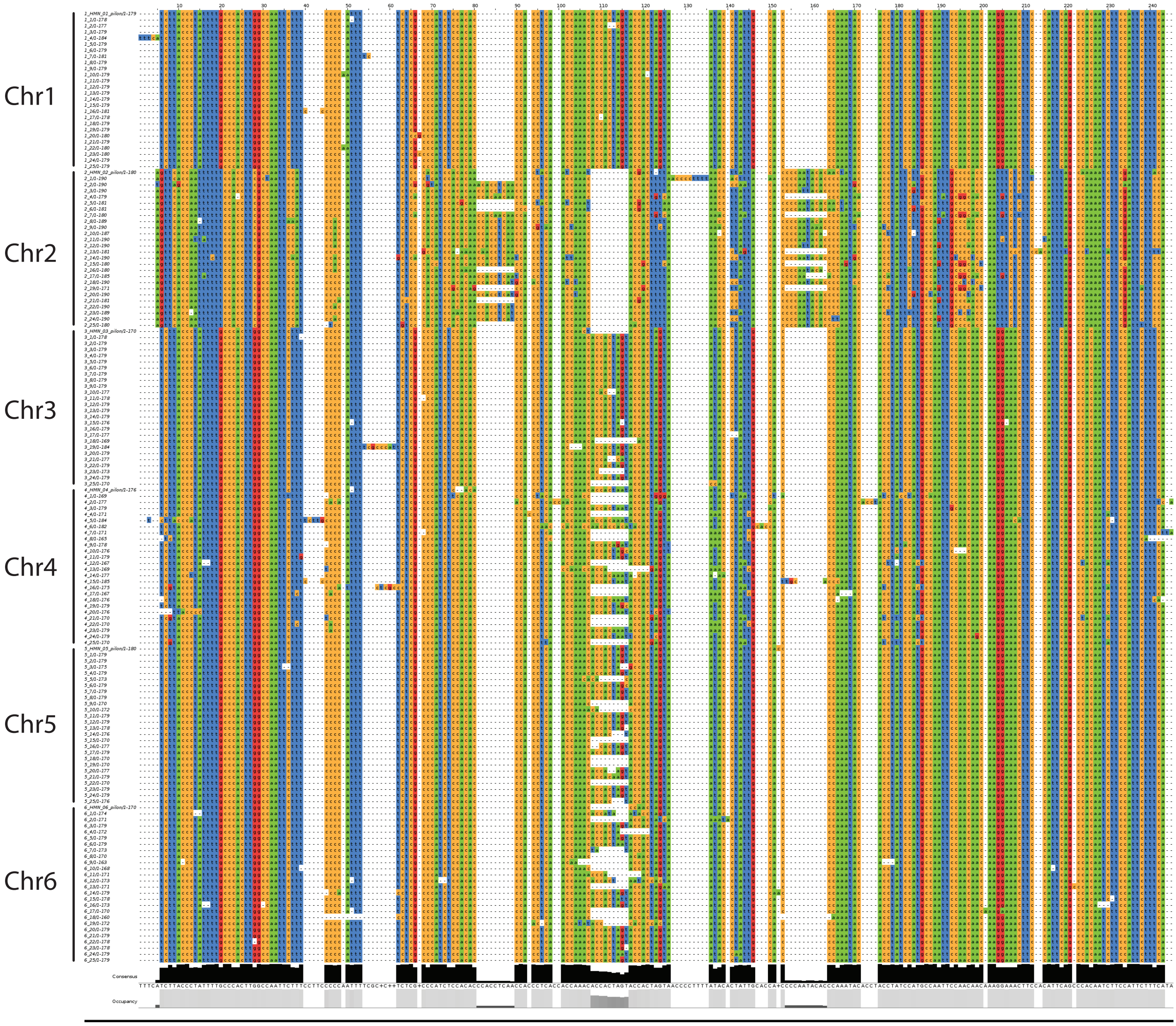

Supplementary Fig. S9. Multiple alignment of the terminal centromeric repeats of each chromosome. 26 consecutive repeat copies were taken from a single location at the end of each of the six chromosomes in turn and aligned in order (top 26 = Chr1, next 26 = Chr2 etc.). Strong conservation of the 179mer centromeric repeat is seen across all chromosomes except Chr2 which shows a second novel repeat type. However, searching within the whole of the Chr2 repeat array shows that the 'canonical' 179mer observed in the other aligned reads is found with 100% coverage and identity. The terminal array on Chr2 is also much larger than those of the other chromosomes and is interspersed with various other repeats not shown here. Full assembly of the Chr2 terminal array is not resolvable without longer sequencing reads. Notably, when the centrosomal repeat arrays are oriented at the same end of each chromosome their sequences are found to be in alignment.
