# Supplementary material for: Complete representation of a tapeworm genome reveals chromosomes capped by centromeres, necessitating a dual role in segregation and protection
Source: BMC Biol. 2020 Nov 9;18:165. doi: 10.1186/s12915-020-00899-w (PMC7653826; doi:10.1186/s12915-020-00899-w)
Supplement: Supplementary file 12 — Additional file 12: Figure S10. The terminal centromeric repeat of chromosome 2. A dotter plot shows that the centromeric repeat not only contains a second dominant repeat motif but is also interspersed with other repetitive elements, unlike the other chromosomes that exhibit a tandem array comprised entirely of the novel 179mer. Within the interstitial sequences we find the top blastx hit to Gag-Pol polyprotein, indicating the centromere has been invaded by transposable elements. [file 12915_2020_899_MOESM12_ESM.pdf]

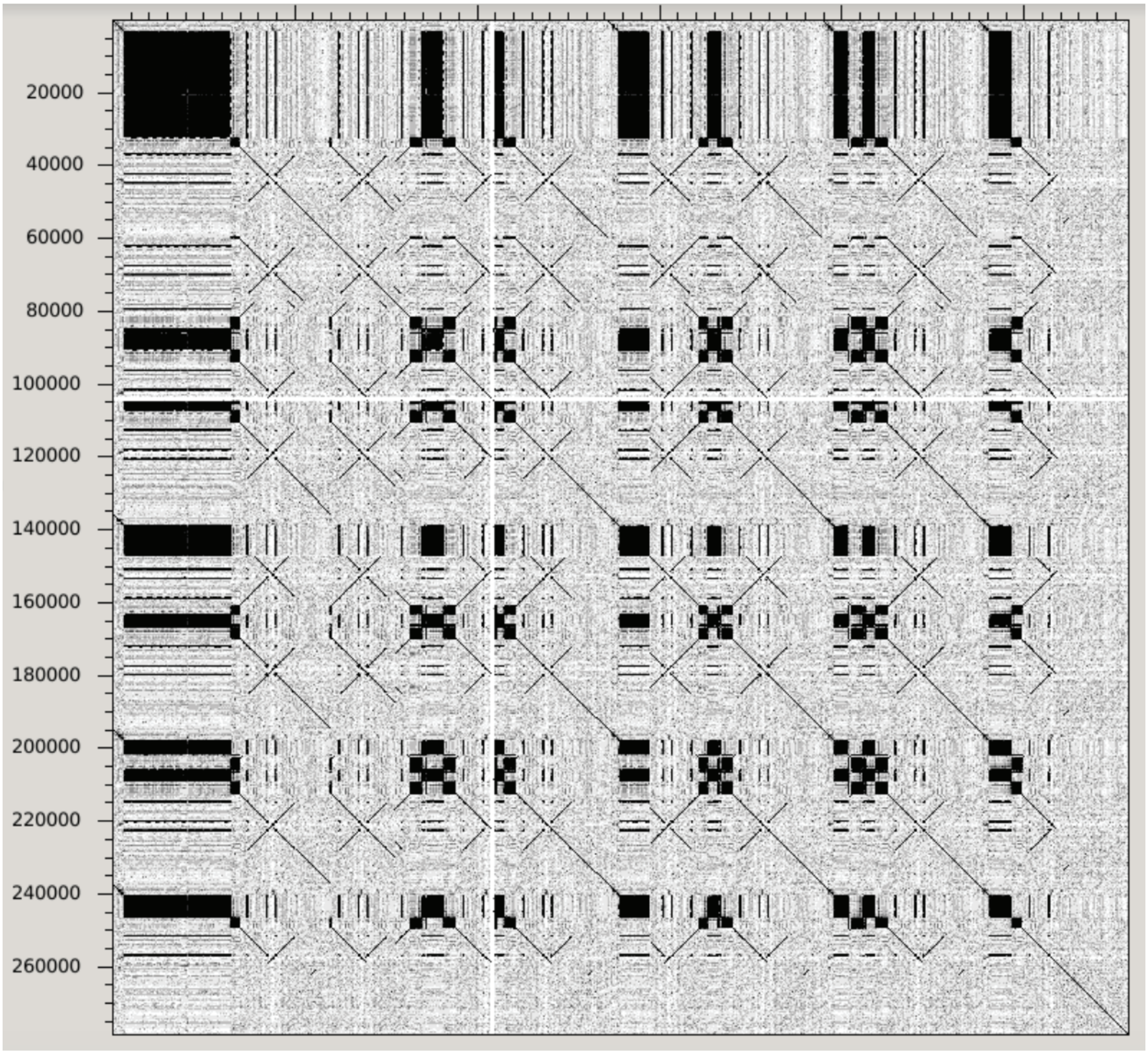

Supplementary Fig. S10. The terminal centromeric repeat of chromosome 2. A dotter plot shows that the centromeric repeat not only contains a second dominant repeat motif but is also interspersed with other repetitive elements, unlike the other chromosomes that exhibit a tandem array comprised entirely of the novel 179mer. Within the interstitial sequences we find the top blastx hit to Gag-Pol polyprotein, indicating the centromere has been invaded by transposable elements.
